# Classification of subtypes and identification of dysregulated genes in sepsis

**DOI:** 10.3389/fcimb.2023.1226159

**Published:** 2023-08-21

**Authors:** Ran Tong, Xianfei Ding, Fengyu Liu, Hongyi Li, Huan Liu, Heng Song, Yuze Wang, Xiaojuan Zhang, Shaohua Liu, Tongwen Sun

**Affiliations:** ^1^General Intensive Care Unit, The First Affiliated Hospital of Zhengzhou University, Henan Key Laboratory of Critical Care Medicine, Henan Engineering Research Center for Critical Care Medicine, Zhengzhou, Henan, China; ^2^Zhengzhou Key Laboratory of Sepsis, Zhengzhou, Henan, China; ^3^Academy of Medical Sciences, Zhengzhou University, Zhengzhou, Henan, China

**Keywords:** sepsis, subtypes, dysregulated genes, biomarker, immune cell infiltration

## Abstract

**Background:**

Sepsis is a clinical syndrome with high mortality. Subtype identification in sepsis is meaningful for improving the diagnosis and treatment of patients. The purpose of this research was to identify subtypes of sepsis using RNA-seq datasets and further explore key genes that were deregulated during the development of sepsis.

**Methods:**

The datasets GSE95233 and GSE13904 were obtained from the Gene Expression Omnibus database. Differential analysis of the gene expression matrix was performed between sepsis patients and healthy controls. Intersection analysis of differentially expressed genes was applied to identify common differentially expressed genes for enrichment analysis and gene set variation analysis. Obvious differential pathways between sepsis patients and healthy controls were identified, as were developmental stages during sepsis. Then, key dysregulated genes were revealed by short time-series analysis and the least absolute shrinkage and selection operator model. In addition, the MCPcounter package was used to assess infiltrating immunocytes. Finally, the dysregulated genes identified were verified using 69 clinical samples.

**Results:**

A total of 898 common differentially expressed genes were obtained, which were chiefly related to increased metabolic responses and decreased immune responses. The two differential pathways (angiogenesis and myc targets v2) were screened on the basis of gene set variation analysis scores. Four subgroups were identified according to median expression of angiogenesis and myc target v2 genes: normal, myc target v2, mixed-quiescent, and angiogenesis. The genes CHPT1, CPEB4, DNAJC3, MAFG, NARF, SNX3, S100A9, S100A12, and METTL9 were recognized as being progressively dysregulated in sepsis. Furthermore, most types of immune cells showed low infiltration in sepsis patients and had a significant correlation with the key genes. Importantly, all nine key genes were highly expressed in sepsis patients.

**Conclusion:**

This study revealed novel insight into sepsis subtypes and identified nine dysregulated genes associated with immune status in the development of sepsis. This study provides potential molecular targets for the diagnosis and treatment of sepsis.

## 1 Introduction

Sepsis is a life-threatening organ dysfunction stemming from host response imbalance to infection. In 2017, 48.9 million septic events were reported worldwide, with 11 million sepsis-associated deaths recorded, accounting for 19.7% of total global deaths (Rudd et al., 2020). In the United States, the costs associated with sepsis treatment amounts to at least $24 billion annually (Liu et al., 2014). Sepsis is a major cause of health concern globally due to its high morbidity, high mortality, and huge economic burden (Weng et al., 2023). The World Health Organization has proposed that member states strive to identify, record and treat sepsis. Sepsis is a heterogeneous syndrome with complex and variable pathophysiological mechanisms, necessitating identification of different clinical biomarkers and phenotypes for precise therapy and optimization of outcomes (Liu et al., 2020; Komorowski et al., 2022).

With the development and wide application of high-throughput sequencing technology, bioinformatics analysis could be used to identify sepsis biomarkers and/or subclasses (Zhao et al., 2021). Zeng et al. found that MAPK14, FGR, RHOG, LAT, and PRKACB were progressively maladjusted in patients with sepsis and septic shock (Zeng et al., 2021). Li et al. found that sepsis-related pathways could be utilized for disease diagnosis, classification, and prognosis (Li et al., 2023). A study based on hierarchical clustering of gene expression profiles of neutrophils and partitioning around medoids was performed to distinguish sepsis subtypes, which were related to sepsis severity (Maslove et al., 2012). Zhang et al. identified two types of sepsis that showed different mortality and reaction to hydrocortisone therapy (Zhang et al., 2020b). Qi et al. explored some mitochondria-related genes to identify the molecular subtypes in sepsis (Shu et al., 2023). At present, there is no research on the classification of sepsis subtypes based on genes expression of differential pathways and validation of the key dysregulated genes in the development of sepsis based on clinical samples.

In addition, the immune response plays a significant role in the pathogenesis and development of sepsis. Immune cells release inflammatory mediators and cytokines aimed at eliminating potential pathogens in the early stage; immune-suppressive mechanisms appear in the late stage during sepsis, resulting in immune dysfunction (Zhang et al., 2023). Bioinformatics research has suggested that SLC2A6 was associated with sepsis-related mortality and correlated positively with infiltration levels of Th1 cells (Li et al., 2021). Research indicated SIGLEC9, TSPO, CKS1B, PTTG3P were significantly associated with infiltration of neutrophils and monocytes during sepsis (Ming et al., 2022). It is of great significance in clinical practice to explore the immune response aspect that is important in the development of sepsis, search key genes associated with immune cells, and elucidate the mechanisms and functions of the molecules and cells involved.

In this research, we analyzed gene expression profiles of sepsis patients in a public database to identify differentially expressed genes (DEGs) and perform enrichment analysis. Gene set variation analysis (GSVA) scores were determined to identify the highest and lowest pathways for dividing sepsis development stages. Short time-series expression miner (STEM) analysis and the least absolute shrinkage and selection operator (LASSO) model were used to identify key genes in sepsis development. We applied the MCPcounter package of R to analyze immune cell infiltration in sepsis. Pearson rank correlation was performed to assay the correlation between key genes and immune cells. Finally, we used clinical samples to validate key genes by reverse transcription quantitative polymerase chain reaction (RT-qPCR). This research not only divided the development stages of sepsis according to differential pathways but also systematically analyzed immune cell infiltration in sepsis. Our findings provide novel ideas for augmenting the diagnosis and treatment of sepsis.

## 2 Materials and methods

### 2.1 Sepsis data source

Two sepsis-related datasets were retrieved from the Gene Expression Omnibus (GEO) database (https://www.ncbi.nlm.nih.gov/geo/). The normalized gene expression matrix was retrieved. We included gene expression profiling based on whole-blood arrays from the GSE95233 dataset (including 102 sepsis patients and 22 healthy controls) and the GSE13904 dataset (including 158 sepsis children and 18 healthy children). All samples collection time point were shown in the **Supplementary Tables 1**, **2**. We applied the stat and dplyr packages in R to annotate and normalize the raw data in these two datasets.

### 2.2 Differential gene expression screening

Differential analysis was performed using the limma package of R to screen DEGs between the sepsis and control groups of the GSE95233 and GSE13904 datasets (Ritchie et al., 2015). DEGs were defined as |log_2_-fold change| > 0.5 and *P* < 0.05. Then, the filtered DEGs from the two datasets were merged to obtain intersection sets. Furthermore, DEGs with the same expression trend (upregulated or downregulated) in the two datasets between the sepsis and healthy control groups were further analyzed.

### 2.3 Enrichment analysis

Multiple pathway datasets (Hallmark, C1-C8 collections) were obtained from Molecular Signatures Database (https://www.gsea-msigdb.org/gsea/msigdb). To explore related biological processes, gene set enrichment analysis (GSEA) was carried out to implement pathway enrichment analysis of upregulated and downregulated genes. *P* < 0.05 was deemed to be statistically significant for enrichment analysis. The results of GSEA were displayed using the fgsea package in R.

GSVA was performed for the enrichment results in the GSVA package of R, and GSVA enrichment scores of each pathway were acquired (Hänzelmann et al., 2013; Yu et al., 2021). Afterwards, we compared GSVA scores between the sepsis and control groups using the limma package in R, so as to identify the upregulated and downregulated pathways in septic patients relative to healthy controls.

### 2.4 Division of development stages in sepsis

Genes belonging to the highest and lowest pathways in the GSVA scores of the two datasets were extracted. Consensus clustering was implemented to select co-expressed genes of the highest and lowest pathways by the ConsensusClusterPlus package in R (Karasinska et al., 2020; Qiu et al., 2021). The parameters were set as follows: reps = 1000, pItem =0.8, pFeature =0.8. Then, median levels of co-expressed genes were calculated to divide sepsis samples of the GSE13904 dataset into four developmental stages.

### 2.5 Short time-series expression miner analysis

We performed STEM analysis of the GSE13904 dataset to cluster the common genes in four developmental stages of sepsis (Ernst and Bar-Joseph, 2006). The clustering module with *P* < 0.05 was defined as a significant clustering module. The significantly clustered genes displayed a more obvious trend of upregulation or downregulation. The genes of the significant module for pathway enrichment analysis were obtained using the GlueGO plug-in of Cytoscape (Shannon et al., 2003).

### 2.6 Model building

We used the LASSO method for dimension reduction analysis of module genes and 5-fold cross validation to extract key genes in sepsis development (Kanwal et al., 2020). Subsequently, we performed logistic regression and random forest methods to evaluate the prediction performance of selected key genes. 5-fold cross validation was conducted to evaluate the prediction efficiency of the LASSO-selected genes. Meanwhile, we calculated the correlation between selected key genes using the circlize package in R.

In addition, we arranged and combined the selected key genes to construct possible logistic regression models. The areas under the receiver operating characteristics curve of each logistic regression model were calculated.

### 2.7 Microenvironment cell populations-counter analysis

Marker genes of immune cell types were acquired as described by Bindea et al. (2013). To estimate immune cell infiltration of samples in the GSE13904 dataset, we used the microenvironment cell populations-counter algorithm with the MCPcounter package in R (Becht et al., 2016). This method stably quantified the abundance of different immunocytes. In addition, Pearson’s correlation analysis was applied to calculate correlations between infiltration levels by different types of immunocytes, as well as correlations between key genes and various infiltration of immunocytes.

### 2.8 RT-qPCR analysis of key genes

#### 2.8.1 Participants

To further validate the potential application of key genes for the diagnosis of sepsis in clinical practice, we selected in-hospital sepsis patients and non-sepsis patients in the intensive care unit (ICU) of the First Affiliated Hospital of Zhengzhou University from June 2022 to December 2022. Patients were excluded if they met any of the following criteria: age < 18 years, ICU stay length < 24 h, pregnancy or lactation, malignancy, receiving immunosuppressive treatment, and AIDS patients. The patients were included in the sepsis group if they met the sepsis 3.0 diagnostic criteria (Singer et al., 2016). The inclusion criteria for the non-sepsis patients were hospitalization in the ICU owing to diseases other than sepsis during the same period. Ethics approval was obtained from the Research and Clinical Trial Ethics Committee of the First Affiliated Hospital of Zhengzhou University (Number: 2021-KY-0467-003). Written informed consent was acquired from all patients or their surrogate decision-makers.

#### 2.8.2 Data collection

Data for the recruited patients, including age, sex, clinical indices such as Sequential Organ Failure Assessment (SOFA) and Acute Physiology and Chronic Health Evaluation II (APACHE II) scores, and laboratory parameters such as white blood cell count, neutrophil percentage, procalcitonin, and C-reactive protein were recorded. Whole blood from patients within 24 h of diagnosis of sepsis or non-sepsis was collected in a 5 ml EDTA tube; red blood cell lysis buffer (Solarbio, Beijing, China) was added, and the sample was fully mixed, followed by centrifugation to isolate nucleated cells for subsequent analysis.

#### 2.8.3 RNA extraction and RT-qPCR

Total RNA of whole blood from enrolled patients was extracted with RNAiso Plus reagent (TaKaRa, Tokyo, Japan) according to the manufacturer’s instructions. A reverse transcription kit (US Everbright, Suzhou, China) was used to generate cDNA as a qPCR template. Quantitative PCR was performed with a Universal SYBR Green qPCR SuperMix kit (US Everbright, Suzhou, China) following the manufacturer’s instructions. RT-qPCR analysis was performed for expression analysis with Applied Biosystems QuantStudio™ 3 (Thermo Fisher Scientific, USA). Relative expression of genes was analyzed by the 2^-ΔΔCt^ method normalized to GAPDH. The primers used were as follows: GAPDH forward: 5’- GTCAAGGCTGAGAACGGGAA-3’, reverse: 5’- AAATGAGCCCCAGCCTTCTC-3’; CHPT1 forward: 5’-AGCTCTTTGACCATGGCTGT-3’, reverse: 5’-TAAGTTCCTAAGCGAGCGGC-3’; CPEB4 forward: 5’- TGAGATCACAGCTAGTTTTCGT-3’, reverse: 5’- TCAATGCATGCATCAATGAGAG-3’; DNAJC3 forward: 5’- TTGGGATGCAGAACTACGGG-3’, reverse: 5’- CCCGAACTTCACTGAGGGAC-3’; MAFG forward: 5’- TGTAGCCCTTGTCTGCACTG-3’, reverse: 5’- CTGTTTTCCCGTGTTCGTTT-3’; NARF forward: 5’- CCGTCGACACTCTGTTTGGA-3’, reverse: 5’- TTGGCCGCATGTCTGAAGAT-3’; METTL9 forward: 5’- TTGGAGCCAACTAGAGGCAG-3’, reverse: 5’- CACTTGCCACCTACGTTTTCC-3’; S100A12 forward: 5’- CGGAAGGGGCATTTTGACACC-3’, reverse: 5’- TCAGCGCAATGGCTACCAGG-3’; S100A9 forward: 5’- TCAAAGAGCTGGTGCGAAAA-3’, reverse: 5’- AACTCCTCGAAGCTCAGCTG-3’; SNX3 forward: 5’- GCTCCCTGGGAAAGCGT-3’, reverse: 5’- GGATGACCAGCGACCTTGTTTA-3’.

### 2.9 Statistical analysis

We used GraphPad Prism version 5.0 (La Jolla, CA, USA) and SPSS version 17.0 (Chicago, IL, USA) for statistical analysis. The median and 25%-75% interquartile ranges were used to assess quantitative variables; categorical variables were described as percentages. Continuous data with a normal distribution were analyzed using Student’s t test, and those with a nonnormal distribution were analyzed with the Mann-Whitney test. Classification data were compared by the chi-square test. We set a two-tailed *P* value < 0.05 as statistically significant.

## 3 Results

### 3.1 Differential expressed genes in sepsis

**Figure 1:**
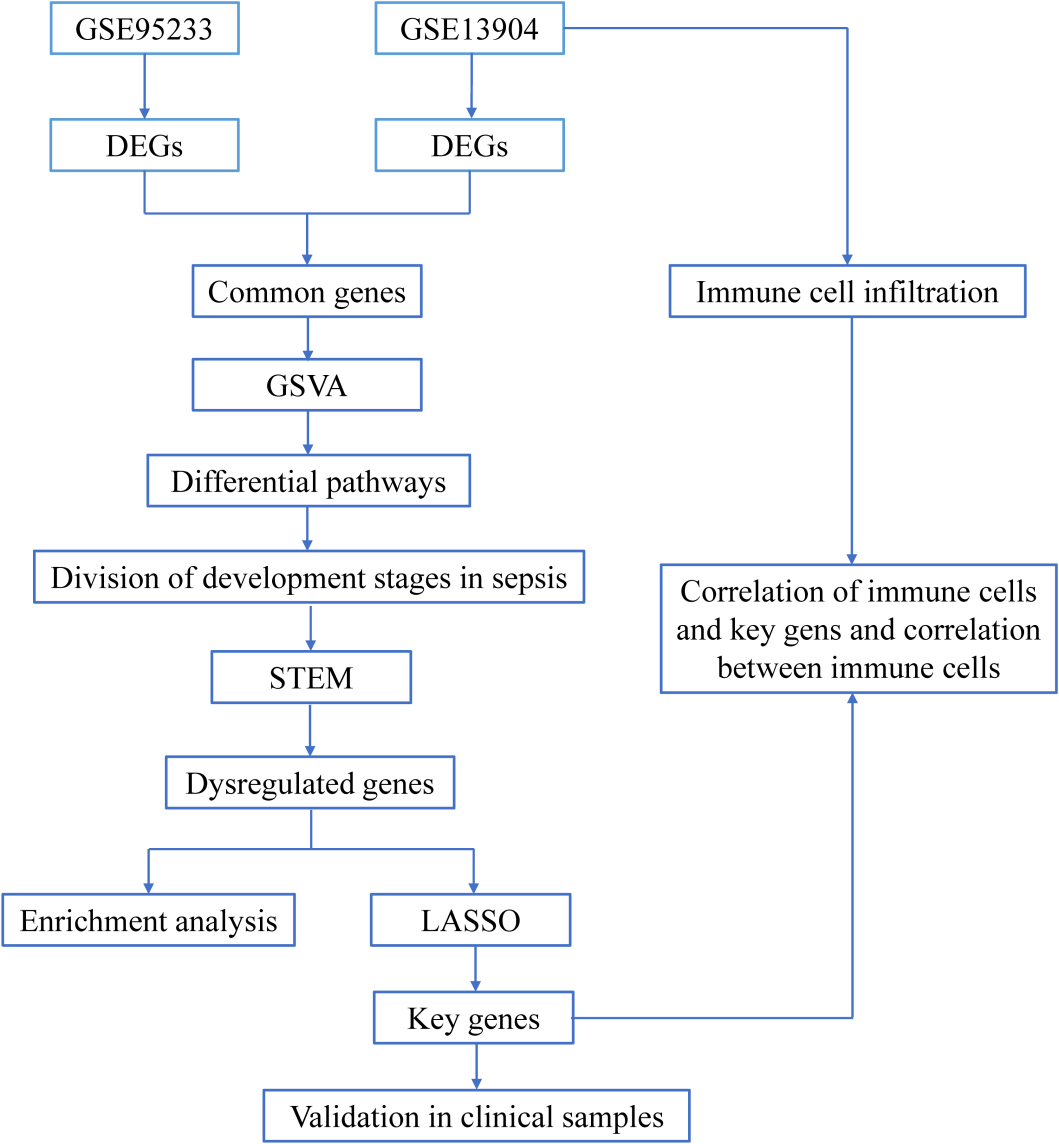
The flow chart of this research. Sequencing data from healthy controls and sepsis samples in the GSE95233 and GSE13904 datasets were analyzed through bioinformatics in order to identify early potential biomarkers of sepsis.

**Figure 2:**
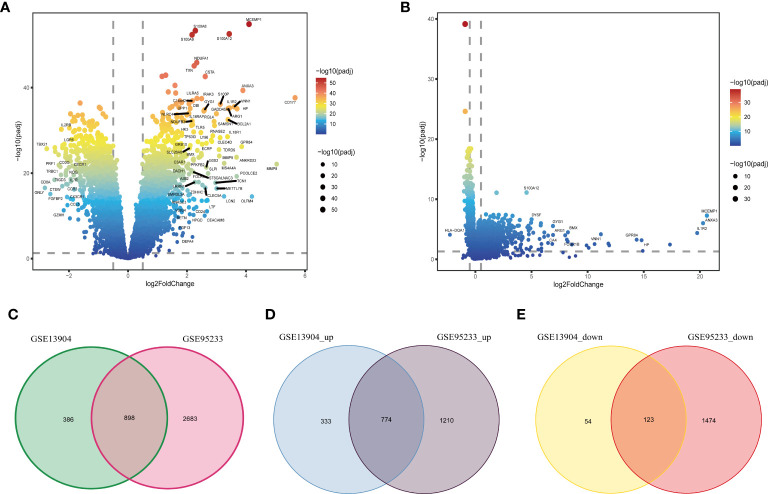
Identification of common DEGs in sepsis. **(A)** The volcano plot of DEGs between the healthy controls and sepsis samples in the GSE95233 dataset. **(B)** The volcano plot of DEGs between the healthy controls and sepsis samples in the GSE13904 dataset. **(C)** Venn diagram of DEGs in the GSE95233 and GSE13904 datasets. **(D)** Venn diagram of up-regulated DEGs in the GSE95233 and GSE13904 datasets. **(E)** Venn diagram of down-regulated DEGs in the GSE95233 and GSE13904 datasets.

**Figure 1** shows the flow chart of our research. We analyzed DEGs between sepsis and control groups to identify genes associated with sepsis. A total of 3581 DEGs in the GSE95233 dataset (**Figure 2A**) and 1284 DEGs in the GSE13904 dataset (**Figure 2B**) were obtained. By comparing DEGs between the two datasets, we observed that 898 genes were common to both (**Figure 2C**). Specifically, we found that 774 DEGs were upregulated (**Figure 2D**) and 123 DEGs downregulated (**Figure 2E**) in the two datasets. These genes at the intersection could be closely related to both adult sepsis and pediatric sepsis.

### 3.2 Functional enrichment of selected genes

**Figure 3:**
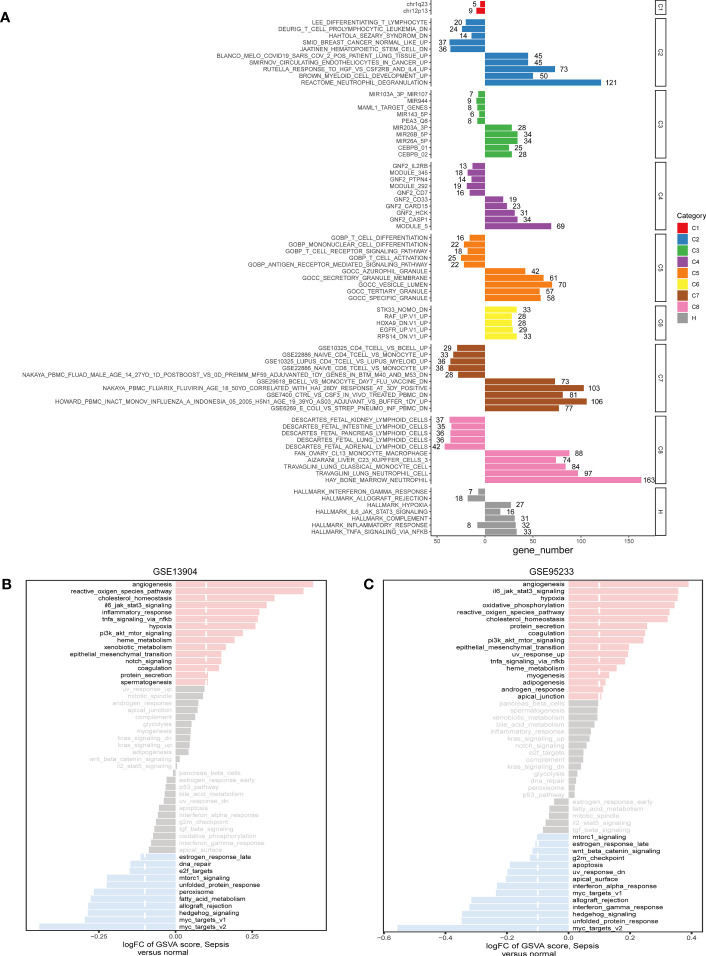
The pathway enrichment analysis of DEGs. **(A)** Significant up-regulated and down-regulated signaling pathways of DEGs between sepsis samples and healthy controls in the C1-C8 and Hallmark collections. The left was down-regulated signaling pathways and the right was up-regulated signaling pathways. **(B)** In the Hallmark collection, significant up-regulated and down-regulated signaling pathways of DEGs between sepsis samples and control samples in GSE13904, as quantified by GSVA. **(C)** In the Hallmark collection, significant up-regulated and down-regulated signaling pathways of DEGs between sepsis samples and healthy controls in GSE95233, as quantified by GSVA. FC, fold change; GSVA, gene set variation analysis.

Based on common DEGs, enrichment analysis results suggested that secretory granule membrane and hallmark hypoxia were upregulated in the sepsis patients compared with the healthy controls but that CD4 T-cell vs. B-cell upregulation, T-cell activation, and Deurig T-cell prolymphocytic leukemia dn were downregulated (**Figure 3A**). These GSEA results revealed the genes upregulated in sepsis to be mainly enriched in metabolism-related pathways; downregulated genes were principally enriched in immune-associated pathways. In addition, we used the hallmark gene set to obtain signaling pathways in which the DEGs between the sepsis and control groups in the GSE95233 and GSE13904 datasets were involved. In the two datasets, angiogenesis was the most obviously highly enriched pathway and myc targets v2 the most obviously less enriched pathway (**Figures 3B, C**).

### 3.3 Identification of four distinct subgroups in sepsis

**Figure 4:**
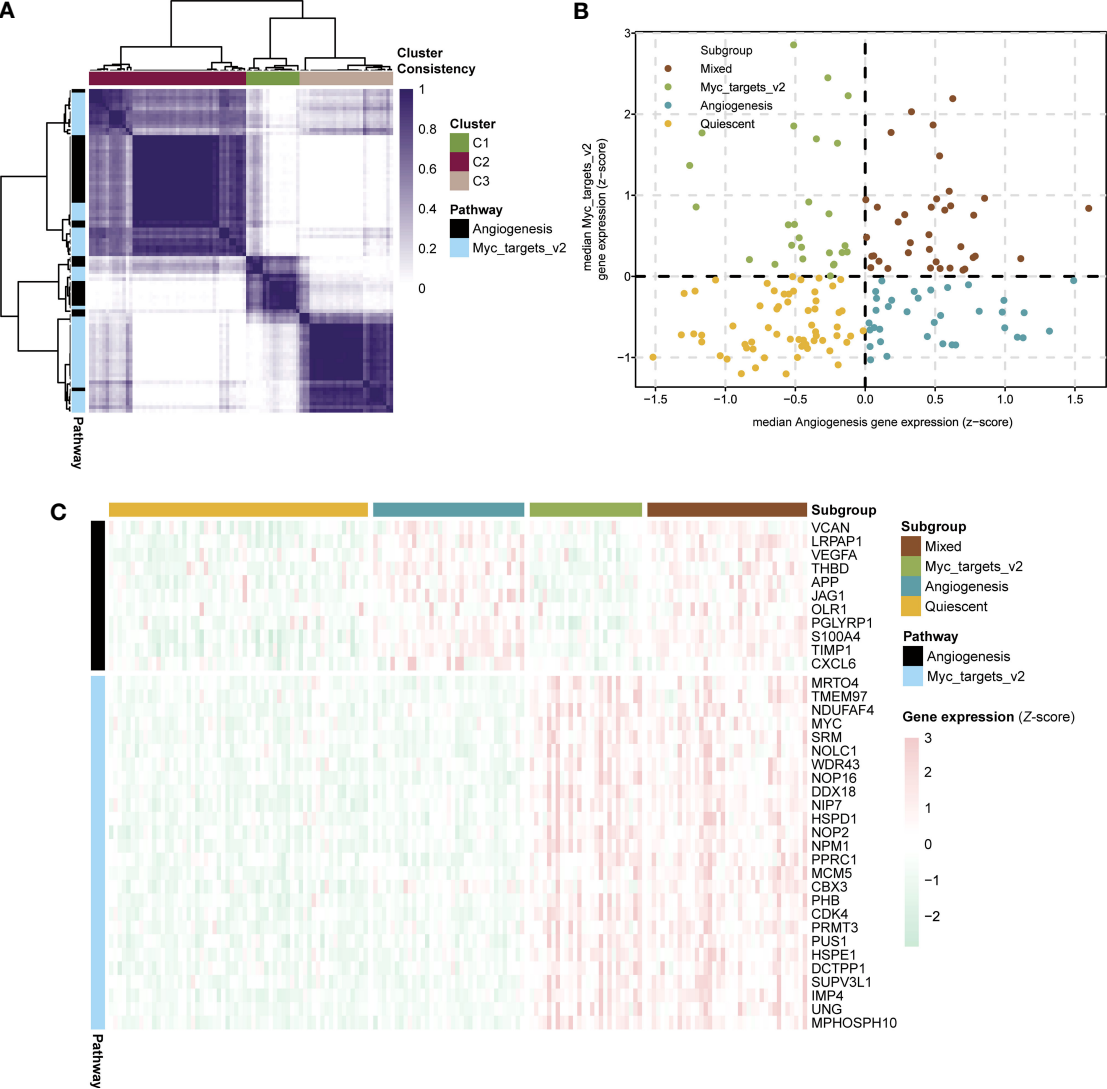
Development stages of sepsis based on expression of angiogenesis and myc targets v2 genes. **(A)** Heatmap showing consensus clustering analysis for angiogenesis and myc targets v2 genes in sepsis samples of GSE13904. **(B)** Scatter plot depicting median expression levels of co-expressed angiogenesis (x-axis) and myc targets v2 (y-axis) genes in sepsis samples of GSE13904. Sepsis subgroups were divided according to the relative expression levels of angiogenesis and myc targets v2 genes. **(C)** Heatmap showing the expression levels of angiogenesis and myc targets v2 co-expressed genes across each subgroup.

To aid in selecting co-expressed genes of the angiogenesis and myc targets v2 pathways and relevant to sepsis biology, we used consensus clustering to obtain robustly angiogenesis (n = 11) and myc targets v2 (n = 26) pathway genes for sepsis subtyping (**Figure 4A**). Besides, we acquired types corresponding to the two pathways (angiogenesis=C2, myc targets v2=C3). On the basis of the median expression levels of co-expressed genes, we assigned the sepsis samples of the GSE13904 dataset to four stages: myc targets v2 (angiogenesis ≤ 0, myc targets v2 > 0), mixed (angiogenesis > 0, myc targets v2 > 0), quiescent (angiogenesis ≤ 0, myc targets v2 ≤ 0), angiogenesis (angiogenesis > 0, myc targets v2 ≤ 0) (**Figure 4B**). The expression levels of angiogenesis and myc targets v2 genes among the four subgroups were visualized in **Figure 4C**. According to the results, we defined the development stages of sepsis in the following order: normal, myc targets v2, mixed-quiescent, angiogenesis.

### 3.4 Key genes associated with sepsis development

**Figure 5:**
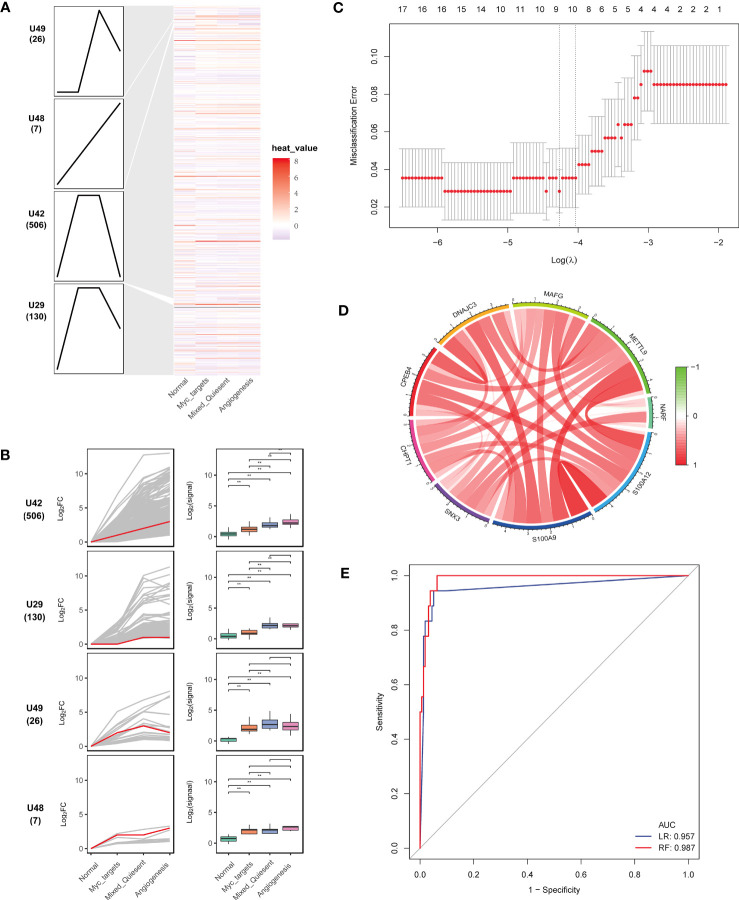
Identification of key genes associated with sepsis development. **(A)** Heatmap of continuously imbalanced genes identified by STEM in sequence: normal < myc targets v2 < mixed-quiescent < angiogenesis. Gene sets were aligned based on cluster distribution to produce simplified expression profiles. **(B)** The box diagrams of STEM genes in four clusters. The line diagrams and box diagrams were applied to show the fold change (log2FC) and absolutely expressed levels, respectively. Highlight representative genes with red lines. **P < 0.01. **(C)** The gene feature selection of optimum parameter (λ) in the LASSO model. **(D)** The correlation circus of key genes expression. Red indicated positive correlation and green indicated negative correlation. The depth of color represented the intensity of correlation. **(E)** The receiver operating characteristic curves of nine key genes by the logistic regression and random forest.

STEM analysis was applied to select genes with persistent imbalance in module genes. In order to screen progressive dysregulation genes during the development of sepsis, we utilized STEM analysis to identify common genes. These common genes fell into four significant clusters (**Figure 5A**). The U42 cluster showed an obvious upregulation trend in the following order: normal < myc targets v2 < mixed-quiescent < angiogenesis (**Figure 5B**). Next, we preformed pathway enrichment analysis of genes in the U42 cluster. The results showed that these genes were mainly enriched in positive regulation of inflammatory response, myeloid leukocyte activation, secretory granule lumen (**Supplementary Figure 1**).

To select the most discriminating genes dysregulated in sepsis development, we used LASSO analysis to obtain nine key genes (CHPT1, CPEB4, DNAJC3, MAFG, NARF, SNX3, S100A9, S100A12, METTL9) from the U42 cluster (**Figure 5C**). These key genes correlated positively with each other (**Figure 5D**). The nine key genes were upregulated in sepsis compared with healthy controls (**Supplementary Figures 2A, B**). The area under the receiver operating characteristics curves of nine genes in the logistic regression and random forest models in the internal validation were 0.957 and 0.987, respectively (**Figure 5E**), which indicated that these nine genes could well predict sepsis development.

### 3.5 Immune cells infiltration in sepsis

**Figure 6:**
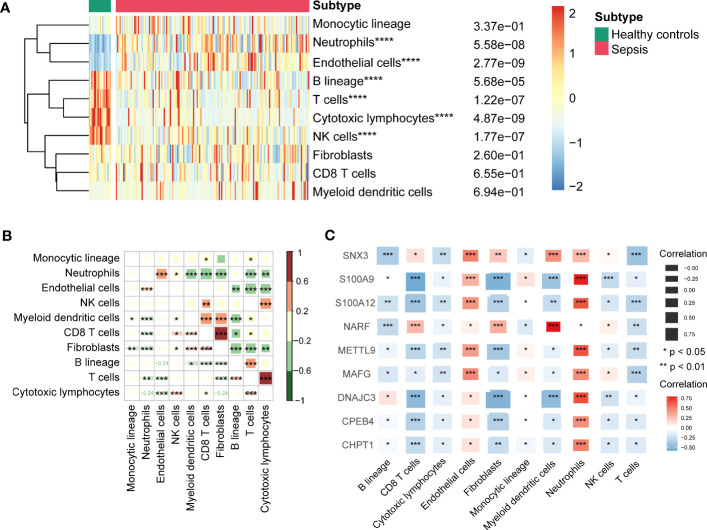
**(A)** Heatmap showing immune cells infiltration between sepsis samples and healthy controls of GSE13904. ****P < 0.0001. **(B)** Correlation between the infiltration levels of immune cells by Pearson’s correlation analysis. Red represented positive correlations and green represented negative correlations. *P < 0.05, **P < 0.01, ***P < 0.001. **(C)** Correlation between key genes and various infiltration of immune cells by Pearson’s correlation analysis. Red represented positive correlations and blue represented negative correlations. *P < 0.05, **P < 0.01, ***P < 0.001.

We identified infiltration of ten immune cell types in GSE13904 by microenvironment cell populations-counter analysis (**Figure 6A**). The results showed that the relative abundances of neutrophils and endothelial cells in sepsis patients were significantly higher than those in healthy patients; the relative abundances of B lineage cells, T cells, cytotoxic lymphocytes, and NK cells were lower. Correlation results between immune cells suggested remarkable positive correlations for fibroblasts and CD8 T cells, cytotoxic lymphocytes and T cells (**Figure 6B**). In addition, we found that these key genes correlated negatively with most types of immune cells, except for endothelial cells and neutrophils (**Figure 6C**).

### 3.6 Validation of key genes expression

**Figure 7:**
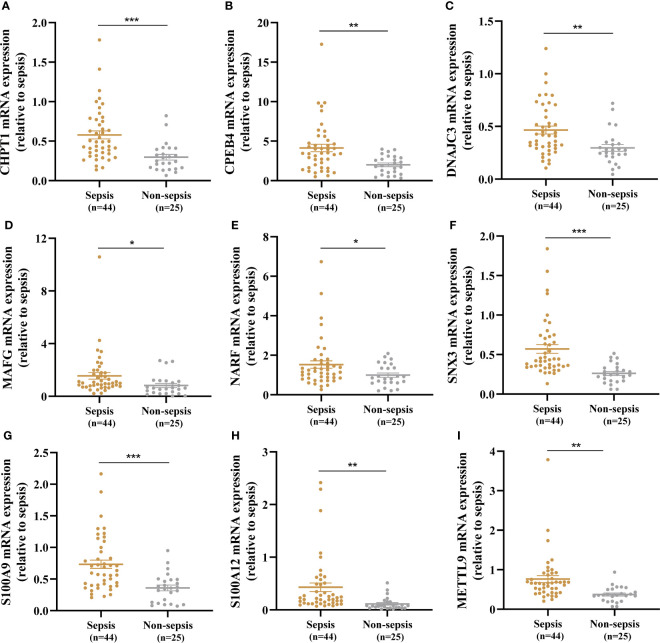
Identification of nine key genes expression in the whole blood between the sepsis and non-sepsis patients. **(A–I)** Expression of CHPT1, CPEB4, DNAJC3, MAFG, NARF, SNX3, S100A9, S100A12, METTL9 between sepsis group and non-sepsis group, respectively. *P < 0.05, **P < 0.01, ***P < 0.001.

A total of 44 sepsis patients and 25 age- and sex-matched non-sepsis patients were enrolled in our research. The baseline demographic characteristics and clinical data between the two groups are summarized in **Supplementary Table 3**. The sepsis patients had significantly higher SOFA and APACHE II scores than the non-sepsis patients on the day of admission to the ICU. The median white blood cell count, neutrophil percentage, procalcitonin level, and C-reactive protein level in the sepsis group were higher than those in the non-sepsis group. In addition, expression of the nine key genes in whole blood between sepsis patients and non-sepsis patients was compared by RT-qPCR, and CHPT1, CPEB4, DNAJC3, MAFG, NARF, SNX3, S100A9, S100A12, and METTL9 levels in the sepsis group were significantly elevated compared with those in the non-sepsis group (**Figures 7A–I**).

## 4 Discussion

Sepsis remains a major public health problem (Tang et al., 2023). In this study, we employed GSVA to identify two differential pathways (angiogenesis and myc targets v2) between sepsis patients and healthy controls. Based on the co-expressed genes of the two pathways, we divided sepsis into four stages: normal, myc targets v2, mixed-quiescent, and angiogenesis. We further used STEM and LASSO analyses to screen nine key genes, CHPT1, CPEB4, DNAJC3, MAFG, NARF, SNX3, S100A9, S100A12, and METTL9, which were progressively dysregulated during the development of sepsis and had important diagnostic functions. More importantly, clinical samples from the ICU verified higher expression of the nine genes in sepsis patients than in non-sepsis patients, which supported the bioinformatics results.

CHPT1 is a key enzyme that participates in the synthesis of glycerophospholipids, catalyzing phosphatidylcholine synthesis and regulating choline metabolism (Wang et al., 2021). Metabolomics analysis demonstrated that serum concentrations of glycerophospholipids and the glycerophospholipid were significantly altered in sepsis (Wang et al., 2021). Our previous research also found that compared with the sham operation group, the plasma metabolites of septic rats involved in the metabolism of glycerol phospholipids and amino acids were significantly changed (Cui et al., 2020). CPEB4, a sequence specific RNA-binding protein, exits in the 3’ untranslated region of some mRNAs (Ivshina et al., 2014). Research showed CPEB4 could stabilize anti-inflammatory transcripts containing AU-rich elements and cytoplasmic polyadenylation elements in the 3’ untranslated region, which was necessary to solve the inflammatory response triggered by lipopolysaccharide (Suñer et al., 2022). Sibilio et al. found that CPEB4 mediated repair and remodeling of acute inflammatory tissue injury (Sibilio et al., 2022). Inflammation and cell stress can result in the accumulation of misfolded or unfolded proteins, known as endoplasmic reticulum (ER) stress (Kim et al., 2013). Similarly, persistent ER stress can also trigger and aggravate inflammation through multiple mechanisms (Zhang and Kaufman, 2008). Research has shown that stress-induced loss of dynamic balance in ER function was closely related to the progression of sepsis (Gong et al., 2022), and DNAJC3 could defend cells against ER stress in response to unfolded proteins (Pauwels et al., 2022). MAFG, a small Maf protein, acts as a heterodimer chaperone with Nrf2 (Li and Zhan, 2022). Sepsis leads to redox imbalance, which is characterized by excessive production of reactive oxygen species. MAFG and Nrf2 were highly involved in the regulation of numerous antioxidant genes at the transcriptional level, and the Nrf2 signaling pathway could respond to excessive reactive oxygen species (Caggiano et al., 2017). NARF is a protein-coding gene that is related to mitochondria and iron-sulfur cluster binding (Ding et al., 2020). Iron-sulfur cluster, a redox regulator, is known for its role in mediating electron transfer in the mitochondrial respiratory chain (Read et al., 2021). Research showed that the iron-sulfur cluster could regulate oxidative stress through superoxide reactive protein, which might affect the progression of sepsis (Kobayashi et al., 2014). SNX3 belongs to the endosomal protein sorting nexin family, which participates in vesicular transport events, including autophagy and endocytosis (Maruzs et al., 2015). Promoting autophagy could alleviate sepsis-related organ injury, which might be a new treatment for sepsis (Sun et al., 2021). S100A9, a proinflammatory alarmin, was upregulated in a variety of infectious diseases (Holzinger et al., 2019; Marinković et al., 2020). A study found that targeting S100A9 could reduce neutrophil recruitment and lung accumulation in sepsis, thereby improving sepsis-related lung injury (Ding et al., 2021). S100A9 usually binds with S100A8 to form heterodimer, which plays an important role in the inflammation (Wang et al., 2018). S100A8/S100A9 heterodimer was reported to help stratify and predict mortality in the septic shock patients (Dubois et al., 2020). Similarly, Davydova et al. suggested that septic shock patients with high levels of S100A12 and S100A8/A100A9 at admission might have a higher risk of death (Dubois et al., 2019). S100A12, similar to S100A9, belongs to the S100 gene family, which plays essential immune response role in inflammation-related diseases (Shah et al., 2017). The research indicated that expression of S100A12 and S100A9 in the peripheral blood of sepsis patients was upregulated compared with that of healthy controls (Uhel et al., 2017), which was consistent with our results. Another study reported that S100A12 promoted inflammation in sepsis-induced acute respiratory distress syndrome by activating the NLRP3 inflammasome signaling pathway (Zhang et al., 2020a). METTL9 is widely specific methyltransferase (Davydova et al., 2021). Daitoku et al. identified that METTL9 catalyzed formation of Nπ-methylhistidine in the S100A9, weakening its affinity for zinc (Daitoku et al., 2021). Given that S100A9 might play an antibacterial role by chelating the zinc necessary for growth of pathogenic bacteria, METTL9 mediated S100A9 methylation may participate in the innate immune response to infection.

Enrichment analysis revealed significant upregulation of metabolic-related pathways and downregulation of immune-related pathways in sepsis. It was reported that sepsis may lead to increased aerobic and anaerobic metabolism, abnormal fatty acid metabolism, and impaired oxygen supply (Ping et al., 2021). PKM2-dependent aerobic glycolysis promoted macrophages stimulated by lipopolysaccharide to release HMGB1 and IL-1β (Xie et al., 2016). Numerous studies have indicated that immunosuppression was a factor for increased susceptibility to mortality and secondary infection in sepsis, and most sepsis-mortality occurred in hypo-inflammation (Gandhirajan et al., 2021; Torres et al., 2022). Severe depletion of dendritic cells might be used to predict sepsis outcome in the early stage (Weber et al., 2015). Besides, sepsis-induced lymphopenia was more prominent in sepsis non-survivors than in survivors (Yao et al., 2022).

Sepsis could damage the innate and adaptive immune responses, which made it impossible to control various types of infections (McBride et al., 2020). Analysis of immune cell infiltration in sepsis patients indicated that most types of immunocyte infiltration decreased, except infiltration by neutrophils and endothelial cells. Due to delayed neutrophil apoptosis, neutrophils level rapidly increased in sepsis (Zhang et al., 2023). The delayed neutrophil apoptosis and formation of neutrophil extracellular traps occurred together with long-term endothelial damage and organ dysfunction (Denning et al., 2019). A significant decrease in lymphocytes (especially CD4 T lymphocytes) was well characterized in sepsis (Martin et al., 2020). In addition, circulating counts of mucosal-associated invariant T cells, natural killer T cells, and gamma delta T cells decreased significantly, the extent of which was related to increased infection risk (Grimaldi et al., 2014). A substantial decline in B-cell counts in sepsis patients was reported, secondary to T lymphocyte deficits and increased apoptosis (Venet et al., 2010). Loss of NK cells directly impacted the immune reaction of sepsis patients (Inoue et al., 2010). Furthermore, the decrease of IFN-γ level owing to accelerated apoptosis of NK cells might increase the possibility of secondary infection in sepsis patients (Wesselkamper et al., 2008). Our research also found that most key genes positively correlated with neutrophils and endothelial cells, and negatively correlated with T cells, CD 8 cells, and NK cells. These findings further supported the potential role of biomarkers and provided new insights for immunotherapeutic targets.

Our research has certain limitations. Firstly, the two databases contain not only adults with sepsis and healthy controls but also children, which may affect biased interpretation of the results. Secondly, we need apply larger clinical samples (adults and children) to verify the expression levels of nine key molecules identified. Further research should explore whether these key genes are associated with the prognosis of sepsis patients.

## 5 Conclusion

In summary, our research revealed that sepsis could be divided into four developmental stages (normal, myc targets v2, mixed-quiescent, angiogenesis). In addition, we screened nine key genes that might be helpful in diagnosing and monitoring the development of sepsis. These key genes showed an inextricable link with the immunological microenvironment in sepsis. This research provides novel insight into latent molecular targets for combating sepsis and promotes a full understanding of the potential immune mechanisms involved in sepsis.

## Data availability statement

The datasets presented in this study can be found in online repositories. The names of the repository/repositories and accession number(s) can be found in the article/**Supplementary Material**.

## Ethics statement

The studies involving humans were approved by The Research and Clinical Trial Ethics Committee of the First Affiliated Hospital of Zhengzhou University. The studies were conducted in accordance with the local legislation and institutional requirements. The participants provided their written informed consent to participate in this study.

## Author contributions

All the authors contributed substantially to the work presented in this article. TS, RT and XD conceived the study. FL, HYL, and HL contributed to data acquisition. HS, YW, XZ and SL contributed to data analysis. TS, RT, and XD contributed to the study protocol and wrote the article. The corresponding author had full access to all of the data and the final responsibility for the decision to submit this article for publication. All authors contributed to the article and approved the submitted version.

## Supplementary material

Supplementary Figure 1 Pathway enrichment analysis network diagram of genes in U42 cluster. Different colors represented different pathways and a circle corresponded to a gene.

Supplementary Figure 2 **(A)** Differential expressed genes between healthy controls and sepsis samples in the GSE13904 dataset. **(B)** Differential expressed genes between healthy controls and sepsis samples in the GSE95233 dataset.
