# Supplementary figures and images for: Classification of subtypes and identification of dysregulated genes in sepsis
Source: Front Cell Infect Microbiol. 2023 Aug 21;13:1226159. doi: 10.3389/fcimb.2023.1226159 (PMC10475835; doi:10.3389/fcimb.2023.1226159)

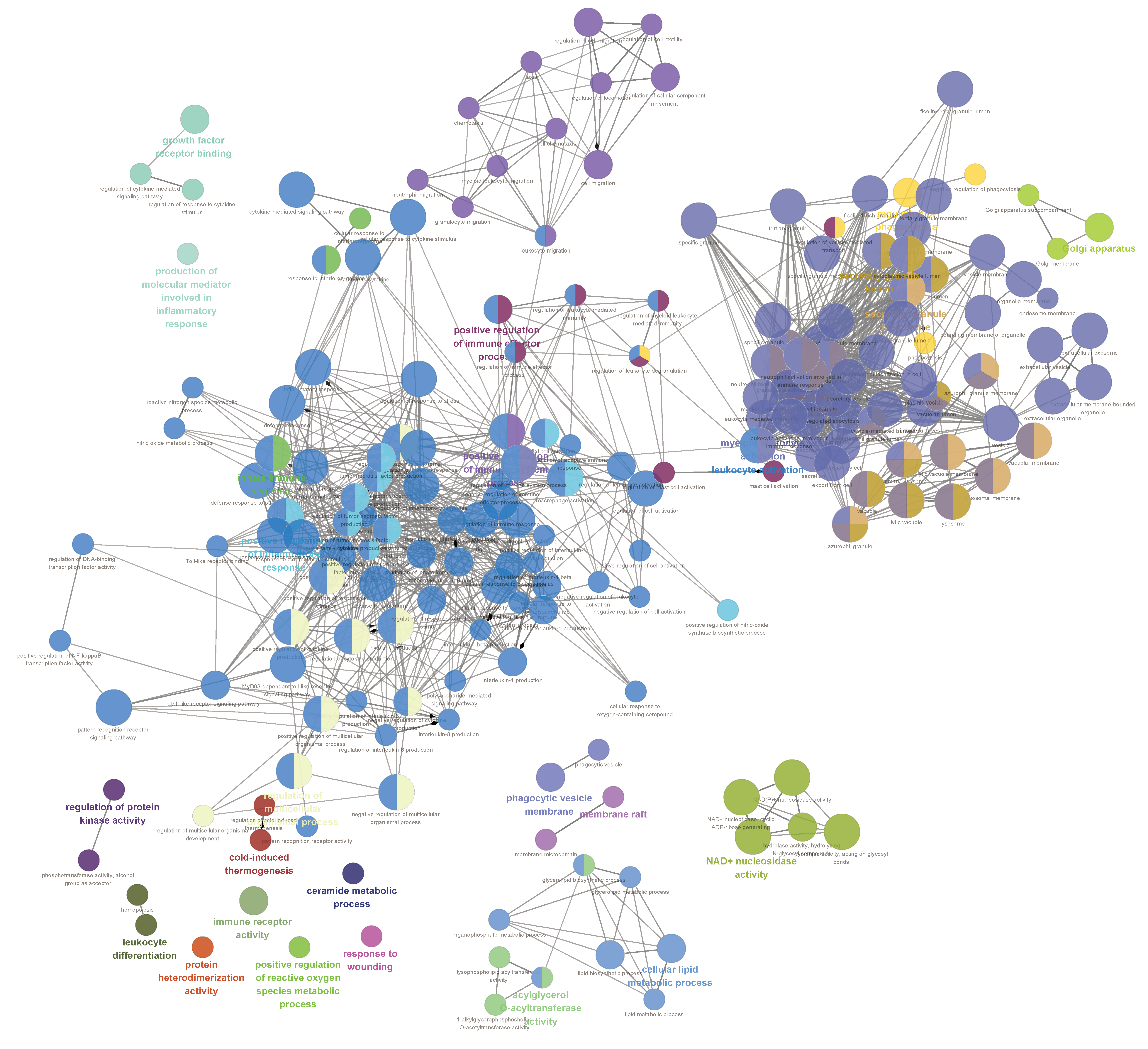

Supplement: Supplementary Figure 1 — Pathway enrichment analysis network diagram of genes in U42 cluster. Different colors represented different pathways and a circle corresponded to a gene. [file Image_1.tif]

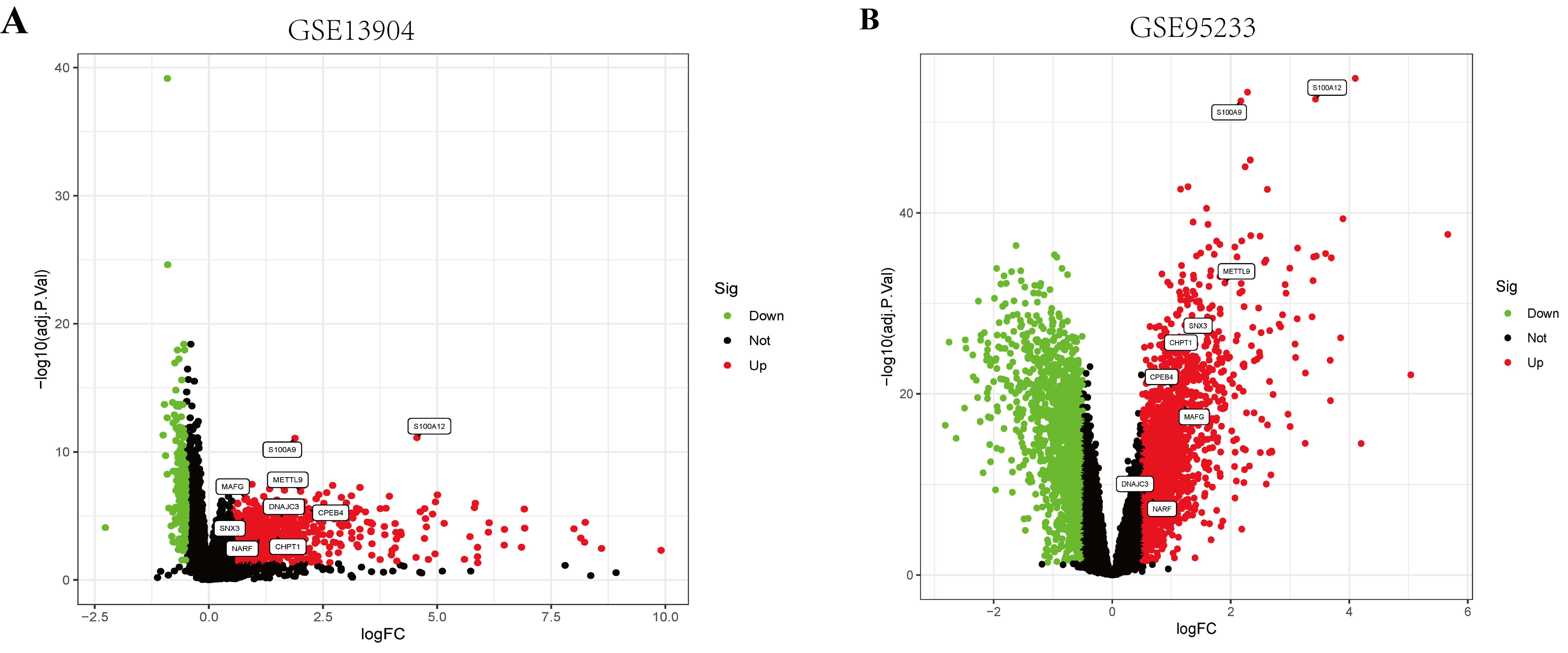

Supplement: Supplementary Figure 2 — (A) Differential expressed genes between healthy controls and sepsis samples in the GSE13904 dataset. (B) Differential expressed genes between healthy controls and sepsis samples in the GSE95233 dataset. [file Image_2.tif]
