# Supplementary material for: Classification of subtypes and identification of dysregulated genes in sepsis
Source: Front Cell Infect Microbiol. 2023 Aug 21;13:1226159. doi: 10.3389/fcimb.2023.1226159 (PMC10475835; doi:10.3389/fcimb.2023.1226159)
Supplement: Supplementary file 3 [file Table_1.docx]

**Supplementary Table 1:** The sample collection time in the GSE13904 dataset.

| **Sample GEO Accession** | **Sample Source Name** | **Sample Collection Time** |
| --- | --- | --- |
| GSM350139 | Control | Day 3 |
| GSM350140 | Control | Day 3 |
| GSM350141 | Control | Day 3 |
| GSM350142 | Control | Day 3 |
| GSM350143 | Control | Day 3 |
| GSM350144 | Control | Day 3 |
| GSM350145 | Control | Day 3 |
| GSM350146 | Control | Day 3 |
| GSM350147 | Control | Day 3 |
| GSM350148 | Control | Day 3 |
| GSM350149 | Control | Day 3 |
| GSM350150 | Control | Day 3 |
| GSM350151 | Control | Day 3 |
| GSM350152 | Control | Day 3 |
| GSM350153 | Control | Day 3 |
| GSM350154 | Control | Day 3 |
| GSM350155 | Control | Day 3 |
| GSM350156 | Control | Day 3 |
| GSM350208 | Sepsis | Day 3 |
| GSM350209 | Sepsis | Day 3 |
| GSM350210 | Sepsis | Day 3 |
| GSM350211 | Sepsis | Day 3 |
| GSM350212 | Sepsis | Day 3 |
| GSM350213 | Sepsis | Day 3 |
| GSM350214 | Sepsis | Day 3 |
| GSM350215 | Sepsis | Day 3 |
| GSM350216 | Sepsis | Day 3 |
| GSM350217 | Sepsis | Day 3 |
| GSM350218 | Sepsis | Day 3 |
| GSM350219 | Sepsis | Day 3 |
| GSM350220 | Sepsis | Day 3 |
| GSM350221 | Sepsis | Day 3 |
| GSM350222 | Sepsis | Day 3 |
| GSM350223 | Sepsis | Day 3 |
| GSM350224 | Sepsis | Day 3 |
| GSM350225 | Sepsis | Day 3 |
| GSM350226 | Sepsis | Day 3 |
| GSM350227 | Sepsis | Day 3 |
| GSM350228 | Sepsis | Day 3 |
| GSM350229 | Sepsis | Day 3 |
| GSM350230 | Sepsis | Day 3 |
| GSM350231 | Sepsis | Day 3 |
| GSM350232 | Sepsis | Day 3 |
| GSM350233 | Sepsis | Day 3 |
| GSM350234 | Sepsis | Day 3 |
| GSM350235 | Sepsis | Day 3 |
| GSM350236 | Sepsis | Day 3 |
| GSM350237 | Sepsis | Day 3 |
| GSM350238 | Sepsis | Day 3 |
| GSM350239 | Sepsis | Day 3 |
| GSM350240 | Sepsis | Day 3 |
| GSM350241 | Sepsis | Day 3 |
| GSM350242 | Sepsis | Day 3 |
| GSM350243 | Sepsis | Day 3 |
| GSM350244 | Sepsis | Day 3 |
| GSM350245 | Sepsis | Day 3 |
| GSM350246 | Sepsis | Day 3 |
| GSM350247 | Sepsis | Day 3 |
| GSM350248 | Sepsis | Day 3 |
| GSM350249 | Sepsis | Day 3 |
| GSM350250 | Sepsis | Day 3 |
| GSM350251 | Sepsis | Day 3 |
| GSM350252 | Sepsis | Day 3 |
| GSM350253 | Sepsis | Day 3 |
| GSM350254 | Sepsis | Day 3 |
| GSM350255 | Sepsis | Day 3 |
| GSM350256 | Sepsis | Day 3 |
| GSM350257 | Sepsis | Day 3 |
| GSM350258 | Sepsis | Day 3 |
| GSM350259 | Sepsis | Day 3 |
| GSM350260 | Sepsis | Day 3 |
| GSM350261 | Sepsis | Day 3 |
| GSM350262 | Sepsis | Day 3 |
| GSM350263 | Sepsis | Day 3 |
| GSM350264 | Sepsis | Day 3 |
| GSM350265 | Sepsis | Day 3 |
| GSM350266 | Sepsis | Day 3 |
| GSM350267 | Sepsis | Day 3 |
| GSM350268 | Sepsis | Day 3 |
| GSM350269 | Sepsis | Day 3 |
| GSM350270 | Sepsis | Day 3 |
| GSM350271 | Sepsis | Day 3 |
| GSM350272 | Sepsis | Day 3 |
| GSM350273 | Sepsis | Day 3 |
| GSM350274 | Sepsis | Day 3 |
| GSM350275 | Sepsis | Day 3 |
| GSM350276 | Sepsis | Day 3 |
| GSM350277 | Sepsis | Day 3 |
| GSM350278 | Sepsis | Day 3 |
| GSM350279 | Sepsis | Day 3 |
| GSM350280 | Sepsis | Day 3 |
| GSM350281 | Sepsis | Day 3 |
| GSM350282 | Sepsis | Day 3 |
| GSM350283 | Sepsis | Day 3 |
| GSM350284 | Sepsis | Day 3 |
| GSM350285 | Sepsis | Day 3 |
| GSM350286 | Sepsis | Day 3 |
| GSM350287 | Sepsis | Day 3 |
| GSM350288 | Sepsis | Day 3 |
| GSM350289 | Sepsis | Day 3 |
| GSM350290 | Sepsis | Day 3 |
| GSM350291 | Sepsis | Day 3 |
| GSM350292 | Sepsis | Day 3 |
| GSM350293 | Sepsis | Day 3 |
| GSM350294 | Sepsis | Day 3 |
| GSM350295 | Sepsis | Day 3 |
| GSM350296 | Sepsis | Day 3 |
| GSM350297 | Sepsis | Day 3 |
| GSM350298 | Sepsis | Day 3 |
| GSM350299 | Sepsis | Day 3 |
| GSM350300 | Sepsis | Day 3 |
| GSM350301 | Sepsis | Day 3 |
| GSM350302 | Sepsis | Day 3 |
| GSM350303 | Sepsis | Day 3 |
| GSM350304 | Sepsis | Day 3 |
| GSM350305 | Sepsis | Day 3 |
| GSM350306 | Sepsis | Day 3 |
| GSM350307 | Sepsis | Day 3 |
| GSM350308 | Sepsis | Day 3 |
| GSM350309 | Sepsis | Day 3 |
| GSM350310 | Sepsis | Day 3 |
| GSM350311 | Sepsis | Day 3 |
| GSM350312 | Sepsis | Day 3 |
| GSM350313 | Sepsis | Day 3 |
| GSM350314 | Sepsis | Day 3 |
| GSM350315 | Sepsis | Day 3 |
| GSM350316 | Sepsis | Day 3 |
| GSM350317 | Sepsis | Day 3 |
| GSM350318 | Sepsis | Day 3 |
| GSM350319 | Sepsis | Day 3 |
| GSM350320 | Sepsis | Day 3 |
| GSM350321 | Sepsis | Day 3 |
| GSM350322 | Sepsis | Day 3 |
| GSM350323 | Sepsis | Day 3 |
| GSM350324 | Sepsis | Day 3 |
| GSM350325 | Sepsis | Day 3 |
| GSM350326 | Sepsis | Day 3 |
| GSM350327 | Sepsis | Day 3 |
| GSM350328 | Sepsis | Day 3 |
| GSM350329 | Sepsis | Day 3 |
| GSM350330 | Sepsis | Day 3 |
| GSM350331 | Sepsis | Day 3 |
| GSM350332 | Sepsis | Day 3 |
| GSM350333 | Sepsis | Day 3 |
| GSM350334 | Sepsis | Day 3 |
| GSM350335 | Sepsis | Day 3 |
| GSM350336 | Sepsis | Day 3 |
| GSM350337 | Sepsis | Day 3 |
| GSM350338 | Sepsis | Day 3 |
| GSM350339 | Sepsis | Day 3 |
| GSM350340 | Sepsis | Day 3 |
| GSM350341 | Sepsis | Day 3 |
| GSM350342 | Sepsis | Day 3 |
| GSM350343 | Sepsis | Day 3 |
| GSM350344 | Sepsis | Day 3 |
| GSM350345 | Sepsis | Day 3 |
| GSM350346 | Sepsis | Day 3 |
| GSM350347 | Sepsis | Day 3 |
| GSM350348 | Sepsis | Day 3 |
| GSM350349 | Sepsis | Day 3 |
| GSM350350 | Sepsis | Day 3 |
| GSM350351 | Sepsis | Day 3 |
| GSM350352 | Sepsis | Day 3 |
| GSM350353 | Sepsis | Day 3 |
| GSM350354 | Sepsis | Day 3 |
| GSM350355 | Sepsis | Day 3 |
| GSM350356 | Sepsis | Day 3 |
| GSM350357 | Sepsis | Day 3 |
| GSM350358 | Sepsis | Day 3 |
| GSM350359 | Sepsis | Day 3 |
| GSM350360 | Sepsis | Day 3 |
| GSM350361 | Sepsis | Day 3 |
| GSM350362 | Sepsis | Day 3 |
| GSM350363 | Sepsis | Day 3 |
| GSM350364 | Sepsis | Day 3 |
| GSM350365 | Sepsis | Day 3 |
