# Supplementary material for: Classification of subtypes and identification of dysregulated genes in sepsis
Source: Front Cell Infect Microbiol. 2023 Aug 21;13:1226159. doi: 10.3389/fcimb.2023.1226159 (PMC10475835; doi:10.3389/fcimb.2023.1226159)
Supplement: Supplementary file 5 [file Table_3.docx]

| **Characteristics** | **Sepsis Patients (n=44)** | **Non-sepsis Patients (n=25)** | ***P* value** |
| --- | --- | --- | --- |
| Age, year, median (IQR) | 57 (46-66) | 59 (54-72) | 0.31 |
| Sex, No. (%) |  |  | 0.98 |
| Male | 29 (65.9) | 16 (64.0) |  |
| Female | 15 (34.1%) | 9 (36.0%) |  |
| Clinical values, median (IQR) |  |  |  |
| White blood cell count, ×10^9^/L | 11.27 (8.13-13.70) | 8.50 (5.75-9.80) | 0.008** |
| Neutrophil percent | 87.80 (82.30-92.80) | 74.80 (66.60-81.50) | 0.000*** |
| PCT, ng/mL | 0.48 (0.22-2.21) | 0.14 (0.07-0.28) | 0.22 |
| CRP, mg/L | 46.46 (8.34-122.9) | 7.66 (5.55-31.85) | 0.006** |
| SOFA score | 5 (4-7) | 1 (0-1) | 0.000*** |
| APACHE II score | 12 (8-21) | 4 (2-14) | 0.77 |

**Supplementary Table 3:** Comparison of general information between sepsis and non-sepsis patients.

Abbreviations: IQR, interquartile range; PCT, procalcitonin; CRP, C-reactive protein; SOFA, Sequential Organ Failure Assessment; APACHE II, Acute Physiology and Chronic Health Evaluation II; * *P* < 0.05，***P* < 0.01，****P* < 0.001.
